# The Development of a Sustainability-Oriented Creativity, Innovation, and Entrepreneurship Education Framework: A Perspective Study

**DOI:** 10.3389/fpsyg.2020.01878

**Published:** 2020-08-13

**Authors:** Yu Shu, Shin-Jia Ho, Tien-Chi Huang

**Affiliations:** ^1^General Education Center, National Taichung University of Science and Technology, Taichung, Taiwan; ^2^Department of Information Management, National Taichung University of Science and Technology, Taichung, Taiwan

**Keywords:** creativity, innovation and entrepreneurship education, education framework, sustainable development goals, higher education

## Abstract

Innovation can include creativity, innovation mechanisms, and entrepreneurship. The ability to innovate is an important indicator of economic and social development, and creativity is an educational indicator of learning effectiveness. This article explores creativity and innovation from an educational perspective and proposes a sustainability-oriented creativity, innovation, and entrepreneurship education framework that uses creative problem solving. This framework contains four layers and three dimensions. The first layer concerns the thinker and basic structure, and the second layer contains the catalyst of sustainable development goals (SDGs). The third layer is the advanced structure of cultivating SDG thinkers. The final layer is the generation of students who will attempt to start up social enterprises. The three aspects apply the creative nature of diffuse thinking to social innovation; apply demand expansion to extend individual needs to societal needs; and apply educational goal development to encourage sustainability. We expect this framework, which can turn thinkers into doers through creativity and social innovation, to apply to different disciplines. This article provides suggestions for (1) designing curriculum in creativity, innovation, and entrepreneurship education (CIE) for different education level and (2) transitioning technical and vocational education in developing economies on the road to sustainable development.

## Introduction

In Taiwan, vocational schools focus more on “doing” than “thinking” and substantially contributed to the country’s economic development from 1960 to 1980. In this context, the tradition of vocational education for “pre-worker” cultivation, which emphasizes convergent skills training instead of divergent thinking, focuses on obedience and discipline rather than leadership and innovation. Seventy years later, Taiwan’s role in the world economy has changed significantly, but the strategies and core values of vocational education have not. The concept of “from thinker to doer” may help update traditional vocational education by shifting the emphasis from a doer’s mastery of skills to a thinker’s inspiration.

Creativity and innovation have been highlighted as essential for the twenty-first century as they drive organizational success in many sectors (Audretsch et al., 2006; Yusuf, 2007; Ferreira et al., 2017). Thinking and doing creatively is crucial for innovations in organizational development. These elements are highly valued in business and educational research, but vocational schools rarely offer curricula for cultivating creativity. Schumpeter (2000) stated that “entrepreneurship as innovation” meant embracing creativity in school education and making innovation and entrepreneurship the goals of creative training; taking these steps could avoid the divergence and impracticality of pure creativity. To start a business, creativity and innovation are critical in every cultural and society. Therefore, for educators, the corresponding question is, Can entrepreneurship be taught?

The demand for enterprise education grew unprecedentedly from the 1980s to the 1990s (Jack and Anderson, 1999), and most entrepreneurship education and training (EET) studies have focused on the benefits of entrepreneurship for economic development (Audretsch et al., 2006; Prieger et al., 2016). Before the 1960s, people did not realize the damage that environmental destruction could do to human development. Now, key issues, such as climate change, loss of biodiversity, frequent disease outbreaks, uneven distribution of food, and increased poverty, are leading people to reflect on the imbalance of nature. Sustainable development is currently regarded as an ark that will carry the essential thoughts of the modern era, such as striving to reverse the currently imbalanced development framework for Earth and pursuing long-lasting peace and prosperity for humankind. Rashid (2019) reviewed EET literature and proposed several findings: (1) little research connects EET literature with sustainable development; (2) EET can advance sustainable development; (3) EET programs that generate environmentally sustainable products are rare; and (4) EET requires innovation. Linking creative entrepreneurship education with Sustainable Development Goals (SDGs) should prove beneficial and practical.

Based on literatures and practical experiences, this study proposes a creativity, innovation, and entrepreneurship (CIE) education framework that should provide a more forward-looking, practical, and realistic direction toward achieving SDGs.

## SDGs and Creativity, Innovation, and Entrepreneurship Education

The former secretary-general of the United Nations (UN), Kofi Annan, stated at the 2002 World Summit on Sustainable Development that “education is the key to achieving sustainable development” (UNESCO, 2005). An important issue for humanity in the twenty-first century, sustainable development should be a crucial core value for entrepreneurs starting their companies and educators planning entrepreneurship education.

However, a review of creativity, innovation, and entrepreneurship (CIE) education studies revealed that most explore the role of pro-market institutions in innovation and entrepreneurship (Gibb, 2002; Sun, 2011; Edwards-Schachter and Wallace, 2017; Li and Yu, 2018; Boudreaux et al., 2019). The value proposition of these studies is economical and profit-oriented. The earliest mention of sustainable development refers to profitable, sustainable management rather than societal sustainability. Overdevelopment, creativity, and innovation that emphasize economic interests may expose humans to more dangerous situations and natural disasters. Compared with the benefit of profit, the merit of sustainable development of the world is vital and inestimable.

**FIGURE 1:**
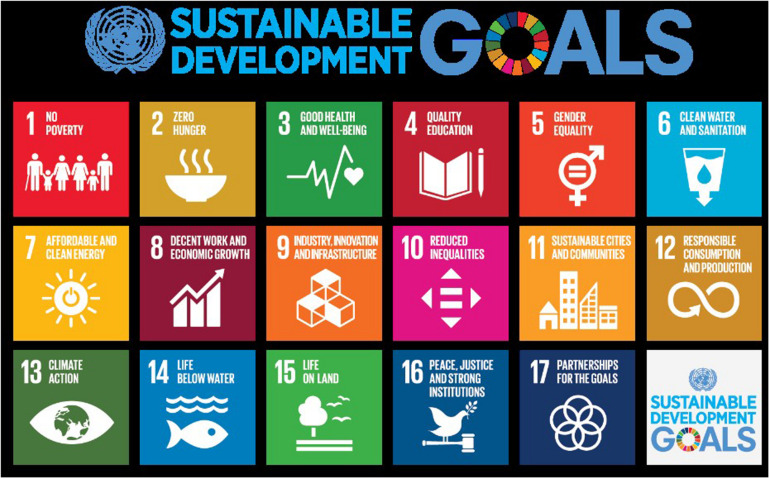
Sustainable development goals, SDGs (United Nations, 2015).

The green movement of the 1980s (Dominick, 1988; Peattie and Ratnayaka, 1992; Jones and Lubinski, 2014) initially focused on the sustainable development of the environment. The Millennium Development Goals released by the UN in 2000 opened the global discussion of sustainably developing society. In 2015, 17 SDGs were offered as guidelines for human development shown in Figure 1 (United Nations, 2015). They are now considered one of the driving forces behind the transformation of private business (Storey et al., 2017).

In turn, the concept of social enterprise has grown more prominent in recent years (Adam, 2004; Galera and Borzaga, 2009; Davies et al., 2019), and social entrepreneurship, a movement about citizen consciousness and voluntarism, has developed. Social entrepreneurship blurs the boundaries between society and business and transforms the notion of non-profit organizations. The implementation of SDGs will not succeed if social enterprise is missing (Pache and Chowdhury, 2012; Lee, 2020). Social enterprises deliver catalytic and innovative participation, which are vital to solving the problems facing humanity. The core idea of social enterprise opposes the logic of mainstream capitalism, which is why it does not emerge naturally. The idea of starting a social enterprise might depend on entrepreneurship education in schools (Tracey and Phillips, 2007; Pache and Chowdhury, 2012).

Therefore, the integration of SDGs into the CIE education framework is imperative. SDGs have five major components: the maintenance of people’s physical and mental health (addressing poverty, hunger, health and well-being, education, and gender equality); the mindset of future social progression (energy, employment, innovation, fairness and justice, and resilient cities); care for the environment (water, oceans, land, consumption and production, and climate change); reflection on institutions; and global cross-regional partnerships. They can provide guidance on the direction of entrepreneurship education and will become important ideas for entrepreneurship education in and of themselves.

## The Sustainability-Oriented Creativity, Innovation, and Entrepreneurship (CIE) Education Framework

### The Four-Layered Framework

**FIGURE 2:**
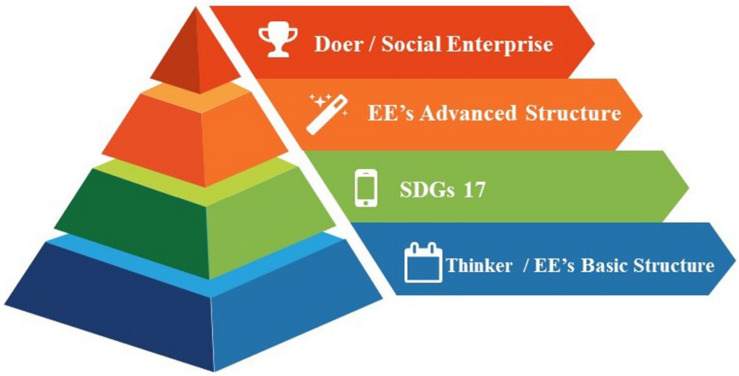
Layers of the proposed CIE education framework (side view).

This paper proposes a sustainability-oriented creativity, innovation, and entrepreneurship (CIE) education framework based on the findings of a literature review and analysis. The researchers aimed to lay the foundation for structural and conceptual development in the process of building each layer of the framework. Along with using “thinker to doer” as the development direction of education, entrepreneurship education is divided into basic and advanced structures. The first level concerns the basic structure of cultivating thinkers through entrepreneurship education. The catalyst of SDGs comprises the second layer, empowering and upgrading the content of the basic structure. The third layer, the advanced structure, encourages thinkers to care about sustainable development goals. The top level, also the final goal, of the proposed CIE education framework is to produce students who will attempt to start up social enterprises that focus on SDGs and solving problems that human beings around the world are facing. The framework is depicted in Figure 2.

To map out the content of the entrepreneurship education framework, we reviewed the literature (Fiet, 2001; Blenker et al., 2013) on three topics: (a) SDGs and CIE education (Storey et al., 2017; Rashid, 2019); (b) creativity and social innovation (Bisanz et al., 2019); and (c) educational goals. To answer the central question “How do we bridge the gap between current CIE education and SDGs?” next, we present three key aspects of the framework. They are based on “creative innovation,” “demand exploration,” and “educational goal development,” respectively.

### The Three Features of the Sustainability-Oriented CIE Education Framework

#### Creativity for Social Innovation

The originality of creativity refers to the discovery or development of ideas, while innovation, which refers to the application of those ideas within a market setting, can be divided into the categories of technology or business model (Antonites and Van Vuuren, 2005; Li and Yu, 2018). Yusuf (2007) analyzed the process from the macro-scope and found that a culture that is positive toward competition, risk-taking, and wealth accumulation; institutions like venture capital and government funding schemes; and financing R&D together comprise the matrix of creativity that can lead to innovation.

The present study incorporated the concept of sustainable development into the aspect of creativity to propose a pathway for CIE education from creativity to social innovation. Rooted in but differing from the business domain, social innovation is defined as “innovative activities and services that are motivated by the goal of meeting a social need” (Mulgan, 2006) and as a novel solution for the social problem that is sustainable (Phills et al., 2008). According to Candi et al. (2019), the social dimension of innovation can be classified into three streams: not-for-profit, hybrid, and business ethics. In the proposed education framework, instructors are free to choose the position according to their enterprise development support, culture, and perspective on civil society. Where venture capital funds are abundant, teachers can choose a business ethics perspective; where civil society or NPOs are developed, the not-for-profit stream may receive more support.

#### Personal Needs for Social Needs

SDGs highlight the world’s urgent social needs and respond to the vulnerable. However, if CIE teaching is directly based on the needs of the world, it may feel disconnected from the student experience and affect learning motivation. Therefore, according to the ARCS motivation theory (Keller, 1987), “the aspect of needs” will begin by reflecting on students’ “personal needs,” which should be more relatable and interesting for learners. UNESCO argues that enterprise education should “foster self-esteem and confidence by drawing on the individual’s talents and creativity” (UNESCO/ILO, 2006). The learning must start with the individual.

However, solutions that focus solely on individual needs may be difficult to innovate and may negatively impact a sustainable society. For an innovation to be successful, it must understand its customers and markets (Selden and MacMillan, 2006). Despite their ingeniousness, many innovations have no market potential (Arthur, 2007) or do not match mass social needs. CIE students will be guided so that they can extend or modify their personal needs according to the perspective of sustainable development and explore social needs. The goal is to develop solutions that have both social value and market potential.

#### Cross-Domain Learning in Entrepreneurship Education

A comprehensive framework of cognition, affection, and skills is indispensable to education development (Kraiger et al., 1993; Yusuf, 2007). In CIE education, innovation demands diverse sources of knowledge and cross-domain learning (Hunter et al., 2008; Scotney et al., 2019). The present study proposes that in CIE education, various disciplines can conduct cross-domain construction learning based on educators’ professional domains of knowledge and skills. This educational approach corresponds to the requirements and ideas proposed toward the generation of feasible solutions to problems.

### The Instruction Plan: Sustainability-Oriented Creative Problem Solving

In the proposed framework, creative problem solving (CPS) is adopted to activate CIE teaching. Alex Osborn developed CPS in the 1940s. CPS differs from traditional problem solving by focusing on logical analysis and pulling from the problem state to the normal state. CPS proposes solutions for developing concrete and feasible action plans to reach the ideal future state that perfectly fits a vision of sustainable development. CPS has four components: understanding the problem, generating ideas, preparing for action, and planning the methods (Treffinger et al., 2008).

**FIGURE 3:**
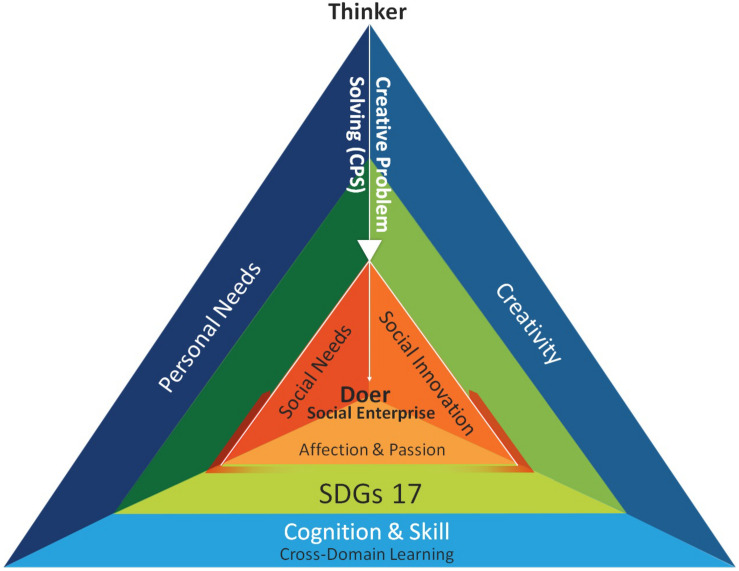
The sustainability-oriented CIE education framework (aerial view).

Figure 3 provides instructors and researchers with an integral understanding of the proposed CIE education framework. This framework is constructed from three aspects and has three levels. While designing a CIE curriculum or a course to cultivate responsible entrepreneurs, instructors can begin with the first level of each aspect. With a CPS teaching approach, educators can train students to move from thinkers to creative doers and encourage their sustainable development thinking.

## Conclusion and Implications

### The Visionary Scenario

Unlike current creativity, innovation, and entrepreneurship education whose aim is business development (Jack and Anderson, 1999; Blenker et al., 2013; Ballor and Claar, 2019), the present study seeks to shape future CIE education by providing an education framework with sustainable human development as its main value.

Although the original intention of this framework originated from the reflection on technical and vocational high school, this framework is a generic CIE education framework, which means that as long as it is related to CIE education, no matter what level of education, teachers can refer to this framework to guide their teaching. According to Bruner, an educational psychologist, “Any subject can be taught effectively in some intellectually honest form to any child at any stage of development” (Bruner, 1960, p. 33). Teachers of different educational levels only need to design CIE courses according to their students’ cognitive development level. The difference in different education levels will only be shown in the complexity of teaching materials, not the focus of teaching. In other words, the connotation of this framework, including sustainable development issues, building social enterprises and cognition, and emotional skills learning, can be used to guide CIE courses at any educational level.

By incorporating the notion of “from thinker to doer” into the learning process, instructors can design or reform courses using the side view (Figure 2) or the aerial view (Figure 3) of the “sustainability-oriented CIE education framework.” This framework provides a conceptual structure for researchers and instructors. The characteristics of its different layers permit variability in instructors’ and students’ processes. Table 1 offers a clarification of the possible role of instructors and students in the different layers of the CIE education framework.

**TABLE 1:** The roles and concepts of the CIE education framework.

**Layers**	**1. Thinker**	**2. 17 SDGs**	**3. Advanced**	**4. Doer social**
	**basic structure**	**(3 stages)**	**structure**	**enterprise**
**Aspects**	Instruction method: CPS			
	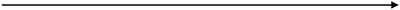			
	Personal			Social
	Benefit/profit			Human development
	Cognitive			Affective

**Roles**				
Instructor	Question Raiser	Guide	Enlightener	Companion
Student	Reflector	Learner	Adjuster	Partner Implementor

From the first layer (Thinker/the basic structure, blue triangle in Figure 3), teachers can guide students with CPS, first on their reflections of individual or personal needs, and then propose solutions with creative thinking strategies. Solutions may include behavioral changes, works, goods, or services. In the process of proposing solutions, students will apply a variety of cross-domain knowledge and skills to evaluate the feasibility and facilitate subsequent production.

There are three stages in the second layer (green triangle in Figure 3). First, “introduction,” the teacher can begin to introduce the 17 SDGs (Figure 1) proposed by United Nations in 2015 one by one through lectures, multimedia, games, or reports. And the depth of exploring the content is determined by the age of the students and the stage of cognitive development. Taking cognitive learning as an example, high school teaching may focus more on understanding poverty issues from the abstract aspects like system, society, economy, politics, etc. (SDGs1: no poverty); while elementary schools focus more on teaching the basic concepts of poverty and from the surroundings of life through observing. Next, “integration” leads students to explore multiple SDGs goals from real and complex social issues. For example, slums are not just about poverty, they are related to at least eight SDGs. The third stage, “sublimation,” based on the understanding of SDGs, from the first stage of creativity, clarification of personal problems and problem-related cognition and technology, choose one or several solvable goals from SDGs, review the solutions developed in the previous step. Students will be inspired to think creatively again, pose appropriate adjustments to “the needs” or “the solutions,” and the problems to be solved by CPS will be revised and sublimated to the third level. In other words, the goal of the entrepreneurship education is to move toward social innovation, focus on social needs, and show affection and enthusiasm for sustainable development, and ultimately guide students to construct social enterprises and conduct social design (Figure 3).

In the third layer (advanced structure, orange triangle in Figure 3), under the affective guidance of the teacher, students will learn about using perspectives on sustainable development, social needs, and social innovation to examine the proposed solutions. Students will feel and appreciate others’ needs based on their understanding of SDGs. From the perspective of social needs, students can think about the market size and customer base for the commercialized product they have in mind. From the perspective of social innovation, they can revise or reframe solutions to achieve sustainable development goals, such as developing countries’ markets, addressing poverty, and caring for seniors.

In the top layer (doer/social enterprise), students will consider themselves partners with and contributors to the world. The teacher could invite them to build projects for the purpose of social innovation or start up a social enterprise.

Unlike previous business-oriented CIE education studies (Jack and Anderson, 1999; Koch et al., 2006; Blenker et al., 2013), this study proposes an entrepreneurial education framework that aims at the sustainable development of the earth and uses social enterprises as a means. Different from past SDGs teaching research, emphasizing the connection of practical field and teaching methods (Fiet, 2001; Edwards-Schachter et al., 2015; Storey et al., 2017; Rashid, 2019), this research includes needs, social innovation, learning objectives, etc., constructing a framework for adjusting current CIE education from multiple angles and aspects.

Society without sustainable development cannot maintain stable socio-economic development. This research proposes a promising educational framework of entrepreneurship (see Figures 2, 3). From the perspective of Society 5.0, this article mentions that the purpose of technology is to solve social problems. Therefore, guided by CPS, social enterprises can be seen as the leading trend of the entrepreneurship education framework of the future. In other words, social design, which is the response to the spirit of building a sustainable global village from the perspective of entrepreneurship education, is the innovative thinking this research provides to on-site teachers under the educational framework of entrepreneurship. With the adjusted courses through different levels of integration, teachers can gradually foster social design talents through entrepreneurship education and provide entrepreneurial talents with a different but sustainable approach to contribute to the sustainability of mankind and the Earth.

### Limitations

Based on a review of the literature, the present study proposed a sustainability-centered entrepreneurship education framework. This proposed educational framework lacks the support of empirical research. It will be developed into a course and will be verified by empirical research in the future.

## Data Availability Statement

All datasets generated for this study are included in the article/supplementary material, further inquiries can be directed to the corresponding author.

## Author Contributions

YS was the first author of this study, conceived of the presented idea, and took the lead in writing the manuscript. S-JH provided the linking between SDGs and entrepreneurship, discussed the relationship between entrepreneurship and the fundamental core ideas of entrepreneurship education. T-CH wrote the two sections: creativity aspect and need aspect, provided critical feedback, and helped shape the research, analysis, and manuscript. All authors discussed the results and contributed to the final manuscript.

## Conflict of Interest

The authors declare that the research was conducted in the absence of any commercial or financial relationships that could be construed as a potential conflict of interest.
